# Reproductive success of the parasitic mite (*Varroa destructor*) is lower in honeybee colonies that target infested cells with recapping

**DOI:** 10.1038/s41598-021-88592-y

**Published:** 2021-04-28

**Authors:** Melissa A. Y. Oddie, Ashley Burke, Bjørn Dahle, Yves Le Conte, Fanny Mondet, Barbara Locke

**Affiliations:** 1 Norwegian Beekeepers Association, Dyrskuev 20, NO-2040 Kløfta, Norway; 2 Department of Ecology, Swedish University of Agricultural Sciences, 750 07 Uppsala, Sweden; 3 INRAE, UR 406 Abeilles et Environnement, Avignon, France; 4 Faculty of Environmental Sciences and Natural Resource Management, Norwegian University of Life Sciences, NO-1432 Ås, Norway

**Keywords:** Conservation biology, Behavioural ecology, Evolution, Sustainability

## Abstract

Cell recapping is a behavioural trait of honeybees (*Apis mellifera*) where cells with developing pupae are uncapped, inspected, and then recapped, without removing the pupae. The ectoparasitic mite *Varroa destructor,* unarguably the most destructive pest in apiculture world-wide, invades the cells of developing pupae to feed and reproduce. Honeybees that target mite infested cells with this behaviour may disrupt the reproductive cycle of the mite. Hence, cell recapping has been associated with colony-level declines in mite reproduction. In this study we compared the colony-level efficacy of cell recapping (how often infested cells are recapped) to the average mite fecundity in *A. mellifera*. Our study populations, known to be adapted to *V. destructor*, were from Avignon, France, Gotland, Sweden, and Oslo, Norway, and were compared to geographically similar, treated control colonies. The results show that colonies with a higher recapping efficacy also have a lower average mite reproductive success. This pattern was likely driven by the adapted populations as they had the largest proportion of highly-targeted cell recapping. The consistent presence of this trait in mite-resistant and mite-susceptible colonies with varying degrees of expression may make it a good proxy trait for selective breeding on a large scale.

## Introduction

Honeybee colonies are highly complex superorganisms that have a wide range of social immunity behavioural traits that can help to protect them from threats like disease and parasite infestations^1,2^. Honeybees are adapted to maximize genetic variability: having the highest genetic recombination rate of any eukaryotic organism, a polyandrous mating system, and haplo-diploid sex determination^3,4^. As a result, they have the potential to adapt to novel threats very quickly. When examining their adaptive potential in depth, it was found that their genetic recombination strategies are geared toward rapid behavioural adaptations in worker bees^5^, which implies a highly flexible adaptive strategy. *Varroa destructor* is a novel parasitic mite parasitizing European honeybees (*Apis mellifera*). It feeds on adult bees and larvae and reproduces in developing brood cells while transmitting viral infections to the point of colony fatality if the mite population is left unchecked^6,7^. This parasite is presently the cause of dramatic colony losses in the commercial sector, and the extreme losses in unmanaged wild and feral populations^8,9^. Over the decades since its introduction, there have been several cases of honeybee populations adapting to regulate parasite levels^10–14^; reviewed by^15^. Often, this adaptation occurs very quickly within the context of evolution. A common observation in these adapted populations is that the mite reproductive output is lower than in regularly managed honeybee colonies. Foundress mites in adapted honeybee colonies entering a brood cell to reproduce during the honeybee’s pupal developmental stage produce fewer viable mated female offspring before the adult bee emerges from the cell. However, the explanation for this lower mite reproductive success in adapted honeybees is unclear. Potentially, adapted traits in honeybees that may emerge to combat this relatively new parasitic threat could likely be behavioural in nature and linked to pre-existing behaviours performed for a broader purpose. Given the complex genetic diversification strategies in honeybees, it stands to reason that all populations have the ability to adapt given there is sufficient selection pressure in the system.

Brood cell recapping is a honeybee behavioural trait involving the opening of a brood cell cap, inspecting the pupae, and then recapping the cell. This behaviour has been recorded in the context of multiple brood diseases^16,17^ and very likely is a general hygienic trait developed to inspect the condition of brood and prevent the spread of infection while maintaining the accuracy of hygienic removal. Brood cell recapping has the potential to disrupt the *V. destructor* reproductive environment and affect the reproductive success by creating hinderances to foundress egg-laying and offspring care, increasing mortality in both male and female mite offspring and potentially hindering subsequent offspring mating. It can be theoretically explained by invoking avoidance behaviour in or increasing risk for foundresses and offspring by introducing changes to the temperature and humidity of the cell environment by exposing brood and mites to open colony air^18,19^. Recently it has been found that cell recapping is a very common trait in European honeybee populations known to survive *V. destructor* without human-mediated treatment^16,20–22^. However, its direct association with reduced mite reproductive success or SMR (supressed mite reproduction) has not been well-examined. We suggest that the potential to develop population-wide mite-targeted cell recapping can be present even in mite-susceptible populations exposed to little or no selective pressure.

Our study is aimed at assessing the efficacy of the recapping trait across colonies in both adapted, mite-surviving populations and non-adapted, mite-susceptible populations. Efficacy in this context refers to the ability of worker bees to detect mite-infested brood cells and target them for uncapping. This is measured by the number of infested cells that display evidence of cell recapping within the total number of infested cells in a sample. Restated: it is the proportion of parasitized cells “affected” by recapping, and will be a measure of both the prevalence and degree of targeting of this trait, two crucial components to the role recapping could play in honeybee mite resistance. We pooled cell recapping data collected from four sources (two surviving populations in France, one in Sweden, and one in Norway along with sympatric, susceptible control populations for each region) and looked at the relationship between the efficacy of this trait (the proportion of infested cells that were recapped) and the average mite reproductive output in each colony, regardless of whether that colony belonged to a surviving or a susceptible population. We also wanted to examine the differences in trait expression (recapping efficacy) in both population types (surviving and susceptible) to better understand the adaptive shift.

## Results

### Mite reproductive success and recapping efficacy

**Figure 1:**
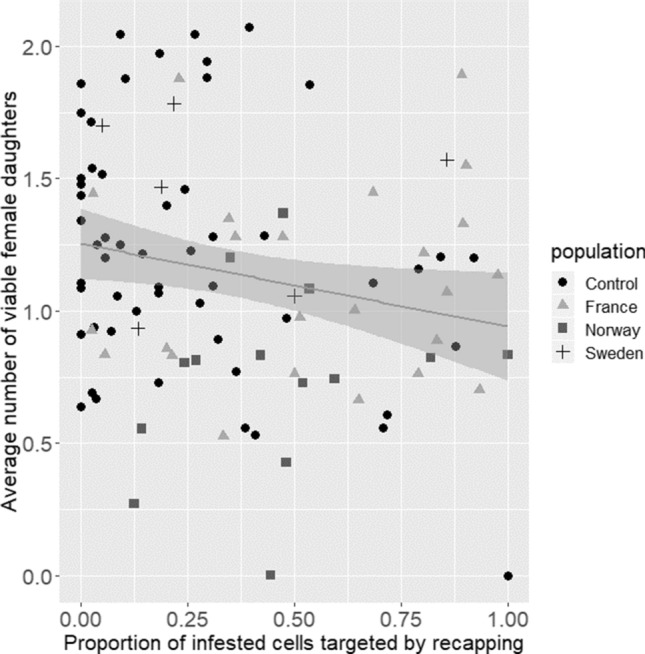
The average number of viable female mite offspring produced per colony in relation to the proportion of infested cells that showed signs of cell recapping. The colonies with lower mite reproductive success tended to have a higher recapping efficacy (glm: χ^2^ = 9.09, df = 1, *p* = 0.003). When mite-surviving and mite-susceptible populations were analysed separately, this pattern was only evident in surviving populations (surviving: χ^2^ = 10.85, df = 1, p = 0.004, susceptible: χ^2^ = 0.32, df = 1, p = 0.59). Bates, D., Maechler, M., Bolker, B. & Walker, S. Fitting Linear Mixed-Effects Models Using lme4. *J. Stat. Softw.* **67**, 1–48 (2015). R Core team. *R: A language and environment for statistical computing*. (R Foundation for Statistical Computing, 2019), https://www.R-project.org.

There was a large amount of variation between colonies (Fig. 1, Table 1, χ^2^ = 299.13, df = 89, *p* < 0.001). Colonies with a lower average mite reproductive success were more accurate with the cell recapping trait and this pattern held true when colonies from control populations were included in this analysis (Fig. 1, Table 1, χ^2^ = 9.09, df = 1, *p* = 0.003).

**Table 1:** Model outputs for factors affecting the average number of female mite offspring per colony in 3225 cells (53 colonies). Significant variables denoted with (*).

Dependent	Parameter	n	df	χ^2^	*p*
Female mite offspring	Brood stage	3225 cells (94 colonies)	5	29.01	< 0.001**
	Number of foundresses		6	16.99	< 0.001**
	Recapping efficacy		1	9.09	0.003*
	Colony		89	299.13	< 0.001**

Foundress number negatively affected the number of offspring produced in cells, as did brood age (Table 1, χ^2^ = 16.99, df = 6, *p* < 0.001, χ^2^ = 29.01, df = 5, *p* < 0.001).

When surviving and susceptible populations were analysed separately, recapping efficacy was only associated with reduced mite reproductive success in surviving colonies (Table 2, surviving: χ^2^ = 10.85, df = 1, *p* = 0.004, susceptible: χ^2^ = 0.32, df = 1, *p* = 0.59, multiple testing accounted for using the Bonferroni correction, α taken to be 0.025). Foundress number and brood stage also had no impact on reproductive success in surviving populations (Table 2, χ^2^ = 2.84, df = 6, *p* = 0.12, χ^2^ = 5.86, df = 5, *p* = 0.027, multiple testing accounted for using the Bonferroni correction, α taken to be 0.025).

**Table 2:** Model outputs for factors affecting the average number of female mite offspring per colony analysed by population. Significant variables denoted with (*).

	Dependent	Parameter	n	df	χ^2^	*p* (0.025)
Surviving population	Female mite offspring	Brood stage	1460 cells (41 colonies)	5	0.12	0.027
		Number of foundresses		6	5.85	0.120
		Recapping efficacy		1	10.01	0.004*
		Colony		39	181.97	< 0.001**
Susceptible population		Brood stage	1742 cells (53 colonies)	5	26.73	< 0.001**
		Number of foundresses		4	18.31	< 0.001**
		Recapping efficacy		1	0.32	0.588
		Colony		52	171.39	< 0.001**

### Cell recapping

The number of foundress mites had a strong impact on recapping probability (Table 2, χ^2^ = 26.19, df = 6, *p* < 0.001). Cells were much more likely to be recapped if the colony was considered part of a surviving population (Table 3, χ^2^ = 18.20, df = 1, *p* < 0.001) and which country the population was from did not appear to affect the rates of recapping (Table 3, χ^2^ = 1.13, df = 2, *p* = 0.57).

**Table 3:** Model outputs for factors affecting the recapping probability in 3225 cells (53 colonies). Significant variables noted with (*).

Dependent	Parameter	n	df	χ^2^	p
Recapped	Brood stage	3225 cells (94 colonies)	5	2.44	0.118
	Number of foundresses		6	26.19	< 0.001**
	Population type		1	18.20	< 0.001**
	Country		89	1.13	0.570

### Population

**Figure 2:**
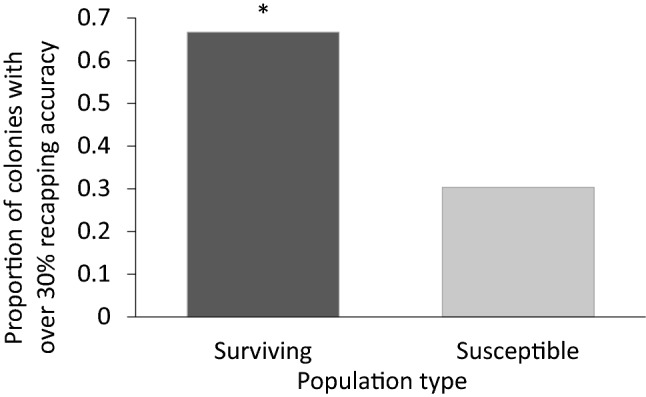
The proportion of colonies with a recapping efficacy of 30% or higher. Proportions were double in colonies that were considered part of a “surviving” population (glm: χ^2^ = 15.05, df = 1, *p* < 0.001). Bates, D., Maechler, M., Bolker, B. & Walker, S. Fitting Linear Mixed-Effects Models Using lme4. *J. Stat. Softw.* **67**, 1–48 (2015). R Core team. *R: A language and environment for statistical computing*. (R Foundation for Statistical Computing, 2019), https://www.R-project.org.

The proportion of colonies with a recapping efficacy of over 30% was over double in the surviving group when compared to the susceptible group (Fig. 2, χ^2^ = 15.05, df = 1, *p* < 0.001, country variation accounted for in model).

## Discussion

Recapping efficacy (the proportion of infested cells that were recapped by bees) is associated with a lower mite reproductive success when mite-surviving and mite-susceptible populations were pooled. This demonstrates the usefulness of cell recapping as an indicator for survivability if not a trait with a direct impact on mite population dynamics. This trait is easily seen in colonies and could be used as an indicator for selective breeding efforts.

The recapping efficacy in mite-susceptible populations was between 22 and 31% and between 35 and 55% in mite-surviving populations. The proportion of colonies with a recapping efficacy over the approximate average of 30% was more than double in surviving populations when compared to susceptible populations. This could indicate enhanced recapping behaviour in the mite-surviving populations is related to a shift in the frequency of a pre-existing trait and not the development of a novel trait^22^. That being said, the level of variation in mite reproductive success and recapping efficacy among all colonies suggests that other traits are at play, that these traits are different between populations and that they have varying levels of expression, likely due to environmental demands and the population dynamics of *V. destructor* in the regions where adaptations occurred.

Depending on whether data on mite-surviving populations and mite-susceptible populations was pooled or not in the analysis, the results were slightly different: The association between this trait and mite reproductive success was not found when susceptible colonies were analysed separately from surviving colonies, though this may be due to the small number of colonies with a high recapping efficacy in the sample. It may also be due to population differences in trait expression and the fact that there are likely multiple traits involved in the adaptation to *V. destructor*. In an “unselected population” like the colonies that are regularly treated to remove the mites (and by extension their selection pressure) there may be a series of traits that have the potential to help colonies persist, but have not yet been “fixed” within the population. Over time, natural selection consistently favours traits that reduce mite reproductive success (like brood removal) and these traits are commonly linked with cell recapping; cell recapping has been found in all mite-surviving populations previously examined for the trait^21,22^ making it a very likely candidate at least by association. Martin et al. found little evidence of this trait in populations naïve to *V. destructor*^22^. It should be noted that even in the susceptible populations of this study, there were a number of colonies that had a high recapping efficacy, and all colonies with recapping accuracies of over 60% had mite reproductive averages of less than 1.3 viable offspring per foundress, which is below the standard average considered by Martin^23^.

The number of offspring decreased with an increasing number of foundress mites in the cell, similarly to previous work on susceptible populations^14,24^. However, in our study this pattern only held for susceptible populations when the two population types were analysed separately. In the surviving populations, neither foundress number nor brood stage seemed to have a significant impact on mite reproductive success, probably because the adapted traits in these populations were stronger, and masking effects of these two variables.

The direct impact of cell recapping remains unclear and several factors impede data collection on the true impact. 1. There may be a selection bias by worker bees that neglect some infested cells where foundress mites produce less offspring naturally (the cell is less active/attractive to worker bees), and target the cells with mites that have a higher reproductive potential, thereby reducing the mite’s reproductive output to the point where no difference can be seen at the end of experimental observation periods. Harris et al.^25^ could not find such discriminations in the choices of workers hygienically removing infested brood, but the potential for worker selectivity in recapping remains to be characterised. 2. Cell recapping could produce a delayed impact on the colony mite infestation. If recapping has a larger disruptive effect on the mating between mite offspring than it does on the egg-laying capacity of the foundress mite, then there may be a generational delay before the full impact of the recapping trait can be seen. Mites often mate more than once within their birth cells, if these events are disturbed, next generation mites would have lower sperm counts in their spermatozoa and their reproductive output would be reduced. As of yet, no experiments have been performed that answer this question directly, however spermatozoa stocks in mated mites tend to increase with multiple mating events ^24^ and low mite sperm counts have been found in another bee population known to survive the parasite^26^. This delayed impact would have an important distinction if the behavioural recapping data was collected during robbing seasons, where horizontal parasite transfer is most likely to occur^27^, and new, fully-mated foundresses were introduced to the colony.

It is also possible that recapping is a derivative of full brood removal within the trait labelled Varroa sensitive hygiene (VSH)^28^. Brood removal would produce the same effect of reduced mite reproductive success as the disruption of the reproductive cycle can hinder subsequent reproductive attempts^29^, however brood removal was examined in at least two of the populations under study (Norway and Sweden) and was found not to exist in surviving colonies at any higher levels than susceptible controls, and the reproductive success of mites was still significantly different^14,30^.

Regardless of direct or indirect impact, the association between cell recapping and reduced mite reproductive success is clear in surviving populations and may only be unclear in susceptible populations due to the low number of colonies displaying the trait. It is possible that survivability is due less to the presence of colonies with good parasite control and more to the absence of colonies with poor parasite control. The presence of unrecorded adaptive traits is quite likely and more research must be conducted to better profile the populations for their optimized survival strategies, as they may differ drastically from one another due to different environmental needs within their unique systems. Cell recapping seems like a general hygienic practice that has been repurposed in the context of *Varroa destructor* and recapping efficacy can be associated with colonies that are able to reduce mite reproductive success to the point of survivability. It can be suggested that tracking the presence and targeting of this trait may lead to more effective breeding strategies to counter *V. destructor*. This trait may also have the potential to manage other brood parasite threats in the future, an example being Tropilaelaps mites (*Tropilaelaps spp.*), which have been assessed as a potential invasive threat^31^. The dynamics of bee behavioural adaptive shifts and responses to novel threats must continue to be studied very carefully.

Due to the large level of variability in the system, as observed in this study, pinpointing the precise effect of cell recapping as well as other mite-resistant traits will require large-scale studies with many colonies under a wide array of environmental conditions and will likely need to last over several years. This size of study may only be possible with tight-knit collaborative efforts between research groups willing to pool their resources for better results. This study will hopefully provide a needed springboard for further such collaborative efforts.

## Methods

### Experimental setup

Colonies were examined during the summer and autumn seasons of their respective countries in 2015, 2016, and 2019. Colonies were sampled from two populations in France (The regions of Avignon and Sarthe), the Gotland “Bond bees” in Sweden, and a commercial population from the Østlandet region of Norway. A total of 53 control colonies (France: 32, Sweden: 6, Norway: 15) and 41 surviving colonies (France: 24, Sweden: 7, Norway: 10) were examined. In order to obtain brood of a similar age, queens were often caged on empty frames for a period of 24–48 h and brood was left in the colonies to be reared until just prior to bee emergence (approximately nine days post capping). One or two frames were chosen from each colony to provide a snapshot of the colony state. Frames were taken from colonies and cells were dissected fresh on the same day or they were frozen for a period of at most one month until the cell dissection could be performed.

### Data collection

Between 150 and 200 cells were dissected on each frame and one frame was sampled per colony. The recapping trait was recorded by inverting each cell cap and looking for the absence of the pupal silk cocoon over a portion of the wax cap seal. In past studies nearly 100% of all uncapped cells were recapped by the bees and thus the trait is not visible from the tops of the cells^21^. The age of the brood was noted and accounted for in the assessment of mite reproductive success as older brood would tend to produce more accurate viable offspring counts. Brood younger than eight days post capping and older than 11 days post capping were not included in the analysis as these brood stages would not permit accurate assessment of mite families. Mite families were extracted carefully with a soft bristle paint brush, and foundresses and offspring (protonymphs, deutonymphs, adult females, and males) were identified using the ontogenic chart published by Martin^23^. Mite reproductive success was determined by counting the average number of viable female mite offspring per foundress. Viable female offspring were identified as offspring that would likely reach a fully moulted stage upon bee emergence and also have been mated by a present male. If there was no male present or if no offspring would reach maturity by the time of bee emergence, the offspring count for that cell was zero.

### Statistical analyses

The statistical analyses were performed using the Lme4 package^32^ in the R statistical analysis program^33^. General linear models were used for all analyses: mite fecundity was analysed using a general linear model, accounting for a quasipoisson error structure and fitted using the Laplace approximation. Colony, foundress number, brood stage, and the recapping efficacy were set as independent variables. Cell recapping was analysed using a general linear mixed effects model, accounting for a binomial error structure (logit function) and fitted using the Laplace approximation. Independent variables included foundress number, brood stage, country of origin, and population type (surviving or susceptible). Colony was set as a random effect to account for between-colony variation. The proportion analysis of the colonies with a recapping efficacy of over 30% was performed using a general linear model accounting for a binomial error structure and fit using the Laplace approximation. Independent variables were set as country of origin and population type. The 30% threshold was chosen based on past observational work showing that there is roughly a 30% decrease in mite reproductive success in mites from surviving populations compared to susceptible populations^13^.

## Data availability

All collected data are provided in the supplementary information or available upon request directed to corresponding authors.
